# Physicochemical and Nonlinear Optical Properties of Novel Environmentally Benign Heterocyclic Azomethine Dyes: Experimental and Theoretical Studies

**DOI:** 10.1371/journal.pone.0161613

**Published:** 2016-09-15

**Authors:** S. M. Afzal, M. A. N. Razvi, Salman A. Khan, Osman I. Osman, Ahmed H. Bakry, Abdullah M. Asiri

**Affiliations:** 1 Physics Department, Faculty of Science, King Abdulaziz University, P.O. Box 80203, Jeddah, 21589, Saudi Arabia; 2 Chemistry Department, Faculty of Science, King Abdulaziz University, P.O. Box 80203, Jeddah, 21589, Saudi Arabia; 3 The Center of Excellence for Advanced Materials, King Abdulaziz University, P.O. Box 80203, Jeddah, 21589, Saudi Arabia; 4 Physics Department, Aligarh Muslim University, Aligarh, 202002, India; 5 Chemistry Department, Faculty of Science, University of Khartoum, P.O. Box 321, Khartoum, 11111, Sudan; Oregon State University, UNITED STATES

## Abstract

Novel heterocyclic azomethine dyes were prepared by the reaction of anthracene-9-carbaldehyde with different heterocyclic amines under microwave irradiation. Structures of the azomethine dyes were confirmed by the elemental analysis, mass spectrometry and several spectroscopic techniques. We studied absorbance and fluorescence spectra of the azomethine dyes in various solvents. They are found to be good absorbers and emitters. We also report photophysical properties like, extinction coefficient, oscillator strength, stokes shift and transition dipole moment. This reflects physicochemical behaviors of synthesized dyes. In addition, their intramolecular charge transfer and nonlinear optical properties, supported by natural bond orbital technique, were also studied computationally by density functional theory. The negative nonlinear refractive index and nonlinear absorption coefficient were measured for these dyes using the closed and open aperture Z-scan technique with a continuous wave helium-neon laser. These are found to vary linearly with solution concentration.

## Introduction

Azomethine (-CH = N) linkages heterocyclic compounds are one of most important compounds in all branches of chemistry such as organic, inorganic, medicinal and material chemistry [1]. They are generally synthesized by the reaction of aromatic aldehydes and aromatic amines [2]. Azomethine compounds are used as intermediate for the various heterocyclic organic syntheses [3]. They are also appropriate in the field of coordination chemistry, forming stable complex with different transition metal [4]. Most of the azomethine compounds are biologically active and these are used as antibacterial, anti-HIV, anti-cancer and anti-inflammatory [5–8]. In the last decades, the azomethine compounds have gained much attention for their wide application in materials chemistry such as nonlinear optical (NLO) materials [9], photonic materials [10], photonic devices [11], optical limiting materials [12], electrochemical sensing [13],light-emitting devices [14], Langmuir films [15] and solar cell materials [16]. Photophysical and physicochemical investigations of the organic dyes namely, oscillator strength, dipole moment, florescent quantum yield, photostability, solvatochromic, piezochromic, and photochemical quantum yield etc. are the important parameters that determine the physical behavior of the dyes having long π-bond conjugation system. These days computational chemistry is considered one of the most important branches of chemistry. It is applied in the calculation of the structures and properties of molecules such as the total energy, frontier molecular orbitals, chemical hardness, electron affinity, ionization energy, nonlinear optical properties and hyperpolarizabilities, using density functional theory (DFT).

Recently there has been great interest of researchers in organic nonlinear optical (NLO) materials. These NLO materials have gained attention due to their application in the fields of optical communication, optical storage, signal processing, harmonic generation, optical switching etc. [17, 18]. Especially, organic materials with extensively delocalized π-electrons have received significant attention due to their large NLO susceptibilities, architectural flexibility and easy fabrication of NLO devices [19–23]. NLO effects in organic molecules are very large as compared to inorganic molecules. This is because they have strong donor-acceptor intramolecular interactions as well as delocalized π-electron systems, in addition to their ability to crystallize in a non-Centro symmetric manner. The nonlinearity in organic dyes is generally governed by the nature of π- bonding sequence and conjugation length. Anthracene-9-carbaldehyde has a good π-bond conjugation system.

In the last few years, microwave has been gradually used in organic synthesis, in comparison to traditional methods. Microwave irradiation method is more convenient, eco-friendly, environmentally benign and easily controlled. A large number of reactions can be carried out at higher yields, in shorter reaction time or under milder conditions of microwave irradiation.

Although much work has been done on azomethine dyes [24–27] on synthesis, characterization, solvatochromism etc.; however, to the best of our knowledge, there is no detailed investigation on their nonlinear optical properties. The long π-bond conjugated system plays an important part in nonlinear optical applications and other areas. The novel dyes being reported here possess much higher nonlinear coefficients and third order susceptibility compared to all the azomethine dyes reported in the literature.

In this paper, we synthesized the azomethine dyes (A1, A2 and A3) by the reaction of anthracene-9-carbaldehyde with different heterocyclic amines under microwave irradiation. Their dipole moments, oscillator strengths, stoke shifts and extinction coefficients were experimentally investigated. However, their gas-phase, total energies, higher occupied molecular orbitals (HOMO), lower unoccupied molecular orbitals (LUMO), chemical hardness (η), chemical potential (μ), electrophilicity constant (ω), natural bond orbitals (NBOs) hyperpolarizabilities were also theoretically studied using CAM-B3LYP/6-31G* level of theory. Nonlinear optical (NLO) properties of the novel heterocyclic azomethine compounds were measured by using the Z-scan technique with a continuous wave (CW) laser.

## Materials and Methods

### Chemicals and Reagents

All the solvents (A.R.) used in this work were of spectroscopic grades. The aldehydes and the different amines were purchased from Acros Organic. 2-Amino-4,5,6,7–tetrahydrobenzo[b] thiophene-carbonitrile was prepared by the method reported by Khan et al [28].

### Apparatus and instruments

A Shimadzu UV-1650 PC spectrophotometer was used for recording UV-Vis electronic absorption spectra with a 1cm Quartz Cell. A rectangular Quartz Cell of dimensions 0.2 cm x 3cm and a Shimadzu RF 5301 PC spectrofluorphotometer was used to record the steady state emission spectra. Tetramethyl silane (TMS) is employed as an internal standard for recording ^1^H-NMR and ^13^C-NMR spectra in CDCl_3_ on a Brucker DPX 600 at 600MHz and 150 MHz spectrometer. Infra-Red (IR) spectra were recorded on a Shimadzu FT-IR 8400S. Melting points of the azomethine dyes were determined with Thomas Hoover capillary melting apparatus.

### Synthesis of azomethine dyes (A1, A2 and A3) by microwave irradiation

Anthracene-9-carbaldehyde (0.0058 mol) and a corresponding active heterocyclic amine (0.0058 mol) in ethanol (99.9%) (25 mL), in the presence of few drops of acetic acid in a beaker (100 mL) were mixed. The reaction mixture was irradiated inside a microwave oven for 3‐5 min. (at 210 Watts, i.e. 30% microwave power). After the completion of the reaction, the reaction mixture was cooled and recrystallized in chloroform and a few drops of distilled ethanol [28]. The details of characterization are available in the supplementary information in S1 File.

### Z-Scan measurement

The nonlinear refractive index (n_2_) and the nonlinear absorption coefficient (β) have been measured by the Z-scan technique, proposed by Sheik-Bahae *et al* [29–31], a very simple, highly sensitive and precise method. It is based on the principle of spatial beam distortion. This technique provides the value of the real and imaginary parts of the third order nonlinear susceptibility as well as the sign of the real part. A convex lens having a focal length of 50 mm focuses tightly a Gaussian profile 10 mW continuous wave He-Ne laser beam. The sample is kept in 1 mm thick rectangular cuvette and is translated along the axis of the laser beam, the Z-direction by a computer controlled stepper motor translation stage at the rate of 1 mm/sec. The details are described in our earlier papers [32,33]. The sample experiences a different incident field at different Z positions during the scan for constant input energy. The transmitted beam through an aperture, kept in the far field, is measured using a photomultiplier tube. The photocurrent is digitized by an A/D converter and the data is stored on a computer. The closed and open aperture scans for the dyes and the pure solvent are recorded, at least three times and averaged, by closing/opening an iris diaphragm. The averaged data for the dyes is divided by the averaged data of the solvent to obtain the normalized transmittance for the dyes.

### Computational Method

The Gaussian 09 suites of programs [34] were used to calculate *ab initio* molecular orbitals for A1, A2 and A3 Dyes. GaussView software [35] was applied for visualizing their geometries and molecular orbitals. The hybrid long-range corrected exchange–correlation Coulomb-attenuating method of the Becke's three parameter Lee-Young-Parr functional (CAM-B3LYP) [36] of the density functional theory (DFT) with double-zeta and polarization and diffuse functions on heavy atoms basis set [6–31+G(d)] has been used to optimize the geometry of the substrates (A1, A2 and A3) to a minimum. The UV-Vis. spectra of A1, A2 and A3 in dimethyl sulphoxide (DMSO), methanol (CH_3_OH) dichloromethane (CH_2_Cl_2_) Tetrahydrofuran (THF), and chloroform (CHCl_3_) solvents were investigated by using Time-Dependent density functional theory (TD-DFT) [37] and the polarizable continuum model (PCM) method [38] applying the CAM-B3LYP/6-31G(d) level of theory.

## Results and Discussion

### Chemistry

**Fig 1:**
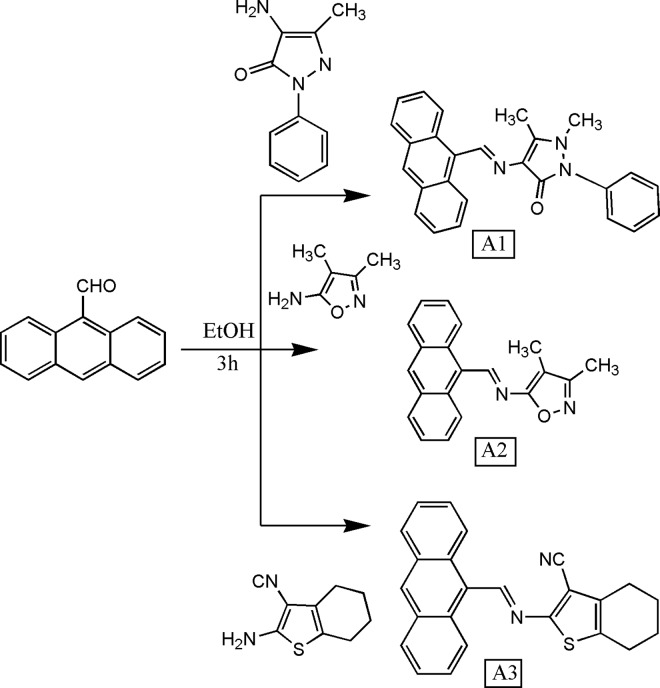
Synthetic route of the compounds A1, A2, and A3.

Azomethine dyes (A1, A2 and A3) were prepared by the reaction of anthracene-9-carbaldehyde with different heterocyclic amines (Fig 1). The newly synthesized azomethine dyes are stable in solution as well as solids. The structure of all the dyes were confirmed by the spectral data GC-MS, FT-IR, ^1^H-NMR, ^13^C-NMR and purity of the dyes was further affirmed by the elemental analysis. The FTIR spectra of azomethine dyes (A1, A2 and A3) showed intense bands at 1557–1580 cm^-1^ for azomethine group (–CH = N–) and at 2917–3027 cm^-1^ due to aromatic C–H. The absence of a band at 1700–1750 cm^-1^ region, has indicated that all–CHO groups are converted to the–CH = N–groups. The ^1^H-NMR spectra provide further signature of the formation of azomethine dyes, from the positional clarification of the protons. Based on the intensity patterns and chemical shifts the assignments of the signals were carried out. The ^1^H-NMR spectra of dyes (A1, A2 and A3) showed sharp singlet at δ 9.76–11.06, confirming the presence of the azomethine (–CH = N–) proton. The appearance of multiplets at δ7.48–9.05 was due to aromatic protons. ^13^C NMR (CDCl_3_) spectra of azomethine dyes (A1-A3) were recorded and the spectral signals are in good agreement with the probable structures. ^13^C NMR spectra showed signals in the range of at 132.63–135.37 ppm and at 114.71–125.64 ppm due to azomethine carbon and aryl carbons respectively.

Finally, the molecular ion peaks from the mass spectra have confirmed the structures of the azomethine dyes. The mass spectra of azomethine dyes A1, A2 and A3 show molecular ion peaks (M‏^+^) m/z of 393, 301 and 368, respectively. All the dyes give analogous fragmentation pattern.

#### Spectral behavior of Azomethine Dyes (A1, A2 and A3)

Figs A to F in S2 File show the absorption and fluorescence spectra of azomethine dyes (A1-A3) at concentration 1 × 10^−5^ M in different protic, polar aprotic and non-polar solvents. The physicochemical properties obtained from steady state absorption and emission spectra are presented in Tables 1–3. As seen in Figs A, C and E in S2 File, polarity of solvent has some effect on the absorption maxima. Dye A1 and A2 are red-shifted with increasing solvent polarity (dye A1 shifted 15 nm and dye A2 shifted 12 nm), on going from n-Hexane to DMSO indicating some polar character of dyes A1 and A2 in the ground state. These features indicate strongly allowed π-π* transitions indicating that the excited state of the dye A1 & A2 is more polar than the ground state. However, dye A3 gives reverse behavior as it is red–shifted with decreasing solvent polarity (A3 shifted 19 nm on going from DMSO to n-Hexane) indicating that the ground state of the dye is more polar than the excited state. These features indicate a strongly allowed n→π* transition [39].

**Table 1:** Spectral data of dye A1 in different solvents.

Solvent	Δ*f*	$E_{T}^{N}$	E_T_ (30) Kcal mol^-1^	λ_ab_ (nm)	λ_em_ (nm)	Ε	*f*	μ_12_ Debye	$\Delta {\bar{\nu }}_{st}$ (cm^-1^)
						M ^-1^cm^-1^			
DMSO	0.266	1.13	67.59	423	446	13100	0.13	3.41	2632
EtOH	0.305	1.18	69.23	410	475	13530	0.18	3.95	3338
MeOH	0.308	1.18	69.23	410	474	14070	0.18	3.95	3293
DMF	0.263	1.15	68.23	419	476	15060	0.17	3.88	2858
CHCl_3_	0.217	1.17	68.89	415	473	14010	0.16	3.75	2955
CH_2_Cl_2_	0.255	1.17	68.72	416	474	13270	0.15	3.63	2941
Acetonitrile	0.274	1.18	69.06	414	475	14290	0.17	3.86	3013
Dioxane	0.148	1.17	68.89	415	473	13570	0.16	3.75	2955
THF	0.208	1.17	68.89	415	472	16300	0.18	3.97	2910
n-Hexane	0.001	1.21	70.07	408	471	15730	0.20	4.15	3278

**Table 2:** Spectral data of dye A2 in different solvents.

Solvent	Δ*f*	$E_{T}^{N}$	E_T_ (30) Kcal mol^-1^	λ_ab_ (nm)	λ_em_ (nm)	Ε	*f*	μ_12_ Debye	$\Delta {\bar{\nu }}_{st}$ (cm^-1^)
						M ^-1^cm^-1^			
DMSO	0.266	1.06	65.12	439	472	12940	0.082	2.76	1593
EtOH	0.305	1.06	65.27	438	469	17410	0.10	3.04	1510
MeOH	0.308	1.08	65.72	435	467	14280	0.081	2.73	1575
DMF	0.263	1.07	65.57	436	468	14010	0.087	2.83	1568
CHCl_3_	0.217	1.08	65.72	435	468	15100	0.097	2.98	1621
CH_2_Cl_2_	0.255	1.09	66.03	433	468	13440	0.092	2.90	1727
Acetonitrile	0.274	1.10	66.49	430	466	14590	0.10	3.01	1796
Dioxane	0.148	1.10	66.64	429	467	14280	0.108	3.13	1897
THF	0.208	1.10	66.64	429	467	15810	0.119	3.28	1897
n-Hexane	0.001	1.11	66.95	427	465	15820	0.121	3.30	1914

**Table 3:** Spectral data of dye A3 in different solvents.

Solvent	Δ*f*	$E_{T}^{N}$	E_T_ (30) Kcal mol^-1^	λ_ab_ (nm)	λ_em_ (nm)	Ε	*f*	μ_12_ Debye	$\Delta {\bar{\nu }}_{st}$ (cm^-1^)
						M ^-1^cm^-1^			
DMSO	0.266	1.06	65.27	438	483	15320	0.13	3.47	2128
EtOH	0.305	1.09	66.33	431	496	13450	0.15	5.70	3040
MeOH	0.308	1.12	67.27	425	497	17710	0.24	4.64	3409
DMF	0.263	1.07	65.42	437	483	15330	0.13	5.89	2180
CHCl_3_	0.217	1.05	64.97	440	493	17080	0.16	3.86	2442
CH_2_Cl_2_	0.255	1.06	65.12	439	485	14730	0.12	3.34	2161
Acetonitrile	0.274	1.09	66.03	433	483	14000	0.13	3.45	2391
Dioxane	0.148	1.08	65.87	434	481	14040	0.12	3.32	2251
THF	0.208	1.00	63.39	451	481	14380	0.079	2.74	1382
n-Hexane	0.001	0.98	62.56	457	481	14370	0.062	2.45	1091

#### Determination of oscillator strength and transition dipole moment

The solvatochromic performances of azomethine dyes (A1-A3) allow us to establish the difference in the dipole moments between the excited singlet and the ground state (Δμ = μ_e_−μ_g_). This variation can be obtained using the simplified Lippert-Mataga equation as follows [39, 40]:

$${\Delta \bar{\nu }}_{st}=\frac{2{\left(\mu_{e}-\mu_{g}\right)}^{2}}{hca^{3}}\Delta f+Const$$ (1)

$$\Delta f=\frac{D-1}{2D+1}-\frac{n^{2}-1}{{2n}^{2}+1}$$ (2)

Where $\Delta {\bar{\nu }}_{st}$ is the Stokes–shift which decreases with decreasing solvent polarity, indicating a weak stabilization of the excited state in non-polar solvents [41]. *Δf* is the orientation polarizability of the solvent, μ_e_ and μ_g_ are the dipole moments in the excited and ground state, respectively, *c* is the speed of light in vacuum, *a* is the Onsager cavity radius and *h* is Planck's constant, n and D are the refractive index and dielectric constant of the solvent respectively, in Eq 2. The Onsager cavity radius was chosen to be 4.2Å because this value is comparable with the radius of a typical aromatic fluorophore [42].

$\Delta {\bar{\nu }}_{st}$, the Stokes shifts of the azomethine dyes (A1-A3) in different solvents were deliberated, as shown in Tables 1–3, using the following equation [39]:

$$\Delta {\bar{\nu }}_{st}={\bar{\nu }}_{ab}-{\bar{\nu }}_{em}$$ (3)

where $\Delta {\bar{\nu }}_{st}$ is the shift in the absorption and emission maxima (cm^−1^).

The Stokes shifts ($\Delta {\bar{\nu }}_{st}$) and the orientation polarizability of the solvent (*Δf*) are used to calculate the change in dipole moments (Δ*μ*) between the excited singlet and ground state from the slope of the plots. The Δ*μ* obtained are as -1.61, -3.03 and 6.43 Debye for azomethine dye A1, A2 and A3, respectively. The negative value for azomethine dye A1 and A2 shows that the excited sate is more polar than the ground state while the positive value for dye A3 shows that the ground sate is more polar than the excited state. The transition dipole moments (μ_12_) between the excited singlet and ground state of azomethine dyes (A1-A3)in various solvents are reported in Tables 1–3, using Eq 4 [43].

$$\mu_{12}^{2}=\frac{f}{4.27\times 10^{-7}\times E_{max}}$$ (4)

where E_max_ is the energy of absorption in cm^-1^ at maximum, and *f* is the oscillator strength.

The oscillator strength *(f)* can be calculated using the following equation:

$$f=4.32\times 10^{-9}\int \varepsilon (\bar{\nu })\;d\bar{\nu }$$ (5)

where *ε* is the extinction coefficient (Lmol^−1^cm^−1^) and $\bar{\nu }$ represents the numerical value of wavenumber (cm^−1^). Oscillator strength values of the azomethine dyes (A1-A3) in various solvents were calculated from the Eq 5 and are listed in Tables 1–3 [44].

#### Fluorescence study with different solvents (A1-A3)

The emission spectra of the 1x10^-5^M of dyes (A1-A3) were measured in various polar aprotic and polar protic solvents and shown in Figs B, D and F in S2 File. Their spectral data are also collected in Tables 1–3. The emission spectra of the dyes **(**A1-A3) in different solvents consist of one broad band. This band can be assigned to S_1_-S_0_ electronic transition. All the compounds give the same behavior *i*.*e*. they show very weak red shifts with increasing solvent polarity (n-Heptane to DMSO) for dye A1 and A2, but A3 (n-Heptane to MeOH) because of excited state hydrogen bonding with alcoholic solvent. Dye A1 shifted 5 nm, dye A2 shifted 7 nm and dye A3 shifted 15 nm indicating the involvement of photoinduced intramolecular charge transfer (ICT) in the singlet excited state than in ground state. The solvent dependence of fluorescence spectra is sometimes called solvatoflurochromism.

The empirical Dimroth polarity parameter, E_T_ and $E_{T}^{N}$ of dyes (A1-A3) were also calculated according to the following equations [45].

$$E_{T}^{N}=\frac{E_{T}\;(solvent)-30.7}{32.4}$$ (6)

$$E_{T}(solvent)=\frac{28591}{\lambda_{max}}$$ (7)

where *λ*_*max*_ corresponds to the peak wavelength (nm) in the red region of the intramolecular charge transfer absorption of the obtained dyes. These are also tabulated in Tables 1–3.

#### Nonlinear Optical (NLO) measurements

**Fig 2:**
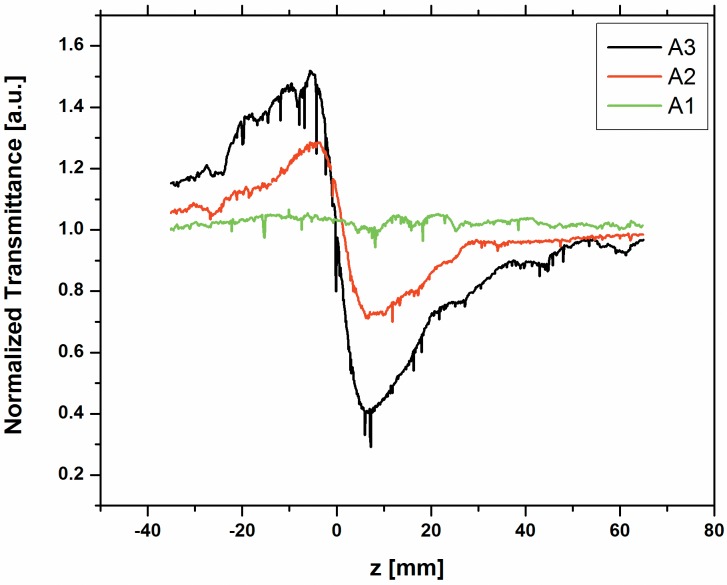
Close-aperture Z-scan signals for the concentration at 100 x10^-5^ M of the three dyes A1, A2 and A3.

We have carried out open aperture and closed aperture Z-scan measurements for the dyes A1, A2 and A3 to estimate nonlinear refractive index and nonlinear absorption. The closed aperture Z-scan experiments are performed by placing an iris aperture in the far field just before the PMT detector. The normalized closed aperture Z-scan curve Fig 2 shows a pre-focal transmittance maximum (peak) followed by a post-focal transmittance minimum (valley) for the dyes A1, A2 and A3 at the concentration 100x10^-5^ M. This is a self-defocusing property of sample and it confirms that the sample has a negative nonlinear refractive index (n_2_). In case of open aperture, the Z-scan data is insensitive to nonlinear refraction and it is expected that the data should be symmetric with respect to the focus. However, the situation is different in the closed aperture case. In closed aperture, due to sample absorption saturation, the peak is enhanced and valley is reduced, which resulted in an asymmetric Z-scan curve about the focus Z = 0 [31].

By least square fitting the normalized transmittance of close aperture scan data with the following equation [46], we obtained the values for the nonlinear refractive index, (n_2_) which are summarized in Table A in S3 File.

$$T(close)=1+\frac{2(-\rho x^{2}+2x-3\rho )}{\left(x^{2}+9)(x^{2}+1\right)}\;\Delta \Phi_{0}$$ (8)

where, $\rho =\frac{\Delta \Psi }{\Delta \Phi_{0}}$; *ΔΦ*_0_ = *kn*_2_*L*_*eff*_*I*_0_ and *ΔΨ* = *βI*_0_*L*_*eff*_/2 are the phase shift due to nonlinear refraction and nonlinear absorption. *x* = *z*/*z*_0_ is related to diffraction length of the beam, *z*_0_, $z_{0}={k\omega }_{0}^{2}/2$; *L*_*eff*_ = [1 − exp(−*α*_0_ *L*)]/*α*_0_, which is effective thickness of the sample, *α*_0_ is linear absorption coefficient, L is the actual thickness of the sample, I_0_ is the on-axis irradiance at the focus I0=2Pπω02.

**Fig 3:**
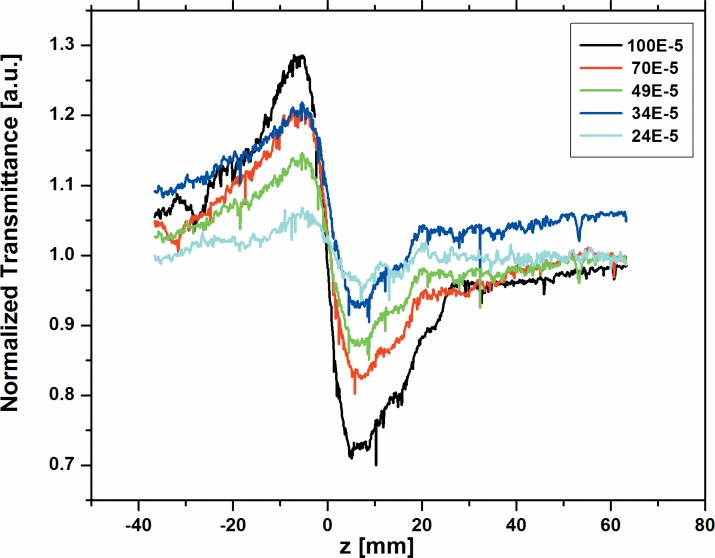
Normalized transmittance for the close aperture of dye A2 at various concentrations.

**Fig 4:**
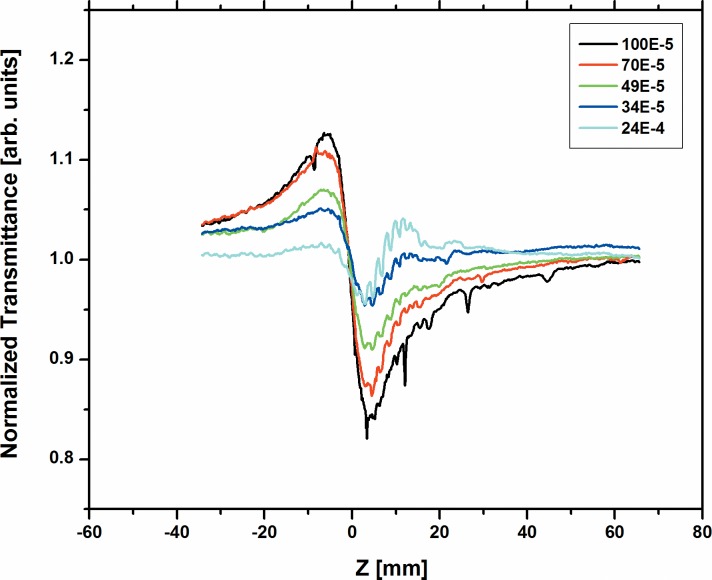
Normalized transmittance for the close aperture of dye A3 at various concentrations.

The nonlinear behavior of all the three dyes namely; A2 and A3 were studied for the various concentrations from 100x10^-5^M to 24 x10^-5^M using 633 nm cw He-Ne laser at 10 mW power. Fig 3 and Fig 4 respectively shows the normalized transmittance for the close-aperture. Dyes A2 and A3 show high negative value of (n_2_) for many concentrations, since A2 and A3 have long л-bond conjugation systems and the nature of the planarity of these dyes is reflected on the high nonlinear refractive index values. However, dye A1 has short л-bond conjugation and less planarity due to the presence of many crowded groups. Hence, we could measure n_2_, β and |*χ*^(3)^| as -1.91x10^-8^ cm^2^/W, 1.22x10^-3^ cm/W and 1.06x10^-6^ esu respectively only at the highest concentration 100x10^-5^M. We could not measure these at lower concentrations, as the nonlinearity for A1 is 20–40 times smaller than A2 and A3 respectively. The values of n_2_ for dyes A2 and A3 at various concentrations obtained from the fit are summarized in Table A in S3 File. Our values reported here are comparable to values reported in earlier papers with cw lasers [47–50].

**Fig 5:**
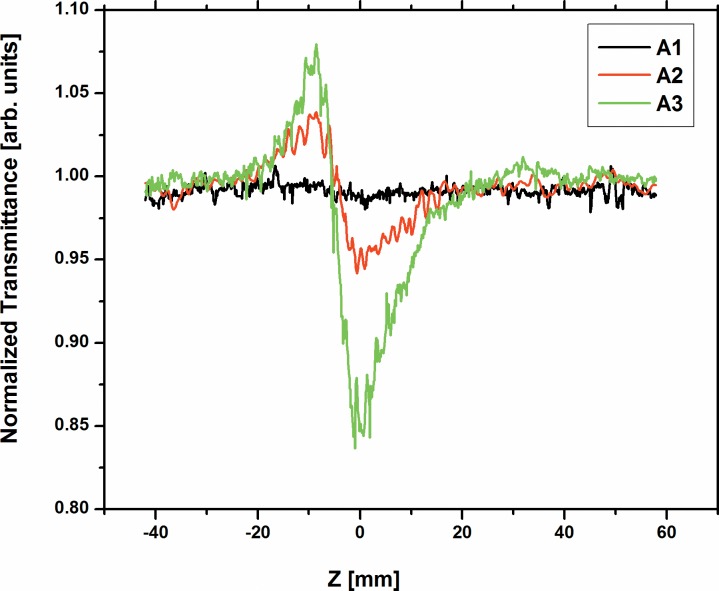
Open-aperture Z-scan signals for the concentration at 100 x10^-5^ M of the three dyes A1, A2 and A3.

**Fig 6:**
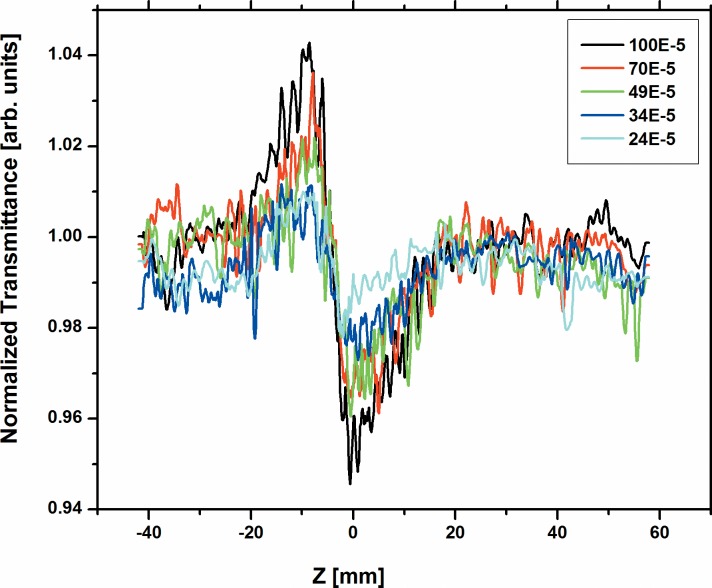
Normalized transmittance for the open aperture of dye A2 at various concentrations.

**Fig 7:**
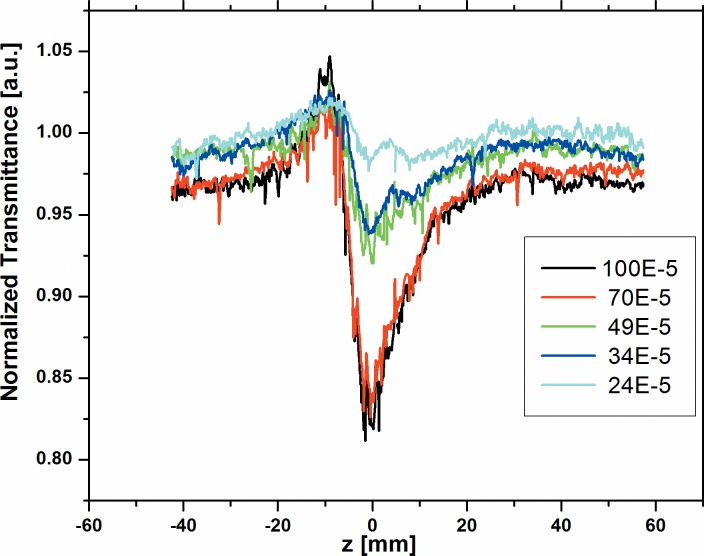
Normalized transmittance for the open aperture of dye A3 at various concentrations.

The open aperture scans for all the three dyes are shown in Fig 5 at concentration 100x10^-5^ M for quick comparison. Fig 6 and Fig 7 shows the open aperture scans of the dyes A2 and A3 respectively at various concentrations.

The nonlinear absorption coefficients β were calculated from the open aperture data by fitting it to the equation [31].

$$T(z,S=1)=\sum_{m=0}^{\infty }\frac{{\left[-q_{0}(z)\right]}^{m}}{{\left(m+1\right)}^{3/2}}$$ (9)

For *q*_0_(0) < 1, where $q_{0}\;(z)=\beta I_{0}L_{eff}/(1+z^{2}/z_{R}^{2})$ and zR=kω022

**Fig 8:**
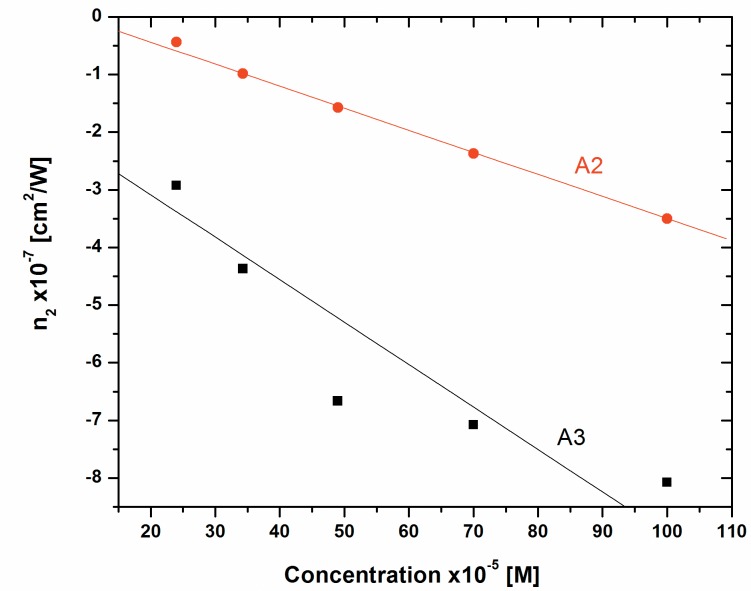
Variation of nonlinear refraction of Dyes A2 and A3 as a function of concentration.

**Fig 9:**
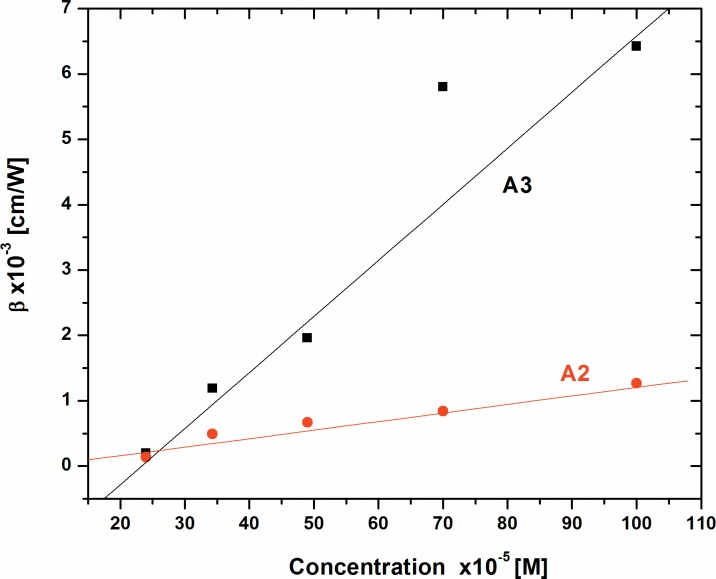
Variation of nonlinear absorption of Dyes A2 and A3 as a function of concentration.

These values are also summarized in Table A in S3 File. The variation of n_2_ and β as a function of concentrations for dyes A2 and A3 are plotted in Fig 8 and Fig 9, respectively. It shows linear variation of these parameters with concentration, this is attributed to the thermal effect. When a highly tight focused beam propagates through these samples, the absorption leads to a spatial variation of temperature in the samples, which ultimately gives a spatial variation of refractive index. This spatial variation acts as thermal lens due to which there is a phase distortion of the laser beam.

It is also observed from the Fig 8 (and Table A in S3 File) that the value of n_2_ for dye A3 is approximately 2.5 times larger than that of the dye A2. However, the nonlinear absorption β is one order of magnitude greater for dye A3 as compared to the dye A2 (Fig 9). The DFT calculations as discussed below also support a similar trend to this observation.

The nonlinear susceptibility is a complex number and is given as:

$$\chi^{\left(3\right)}=\chi_{R}^{\left(3\right)}+i\chi_{I}^{\left(3\right)}$$ (10)

where the real and imaginary parts are related to nonlinear refractive index and nonlinear absorption coefficient, and these were evaluated using the following equations:

$$\chi_{R}^{\left(3\right)}(esu)=10^{-4}\frac{\varepsilon_{0C^{2}n_{0}^{2}}}{\pi }n_{2}\;(cm^{2}/W)$$ (11)

and

$$\chi_{I}^{\left(3\right)}(esu)=10^{-2}\frac{\varepsilon_{0\;C^{2}n_{0}^{2}\lambda }}{{4\pi }^{2}}\beta \;(cm/W)$$ (12)

**Fig 10:**
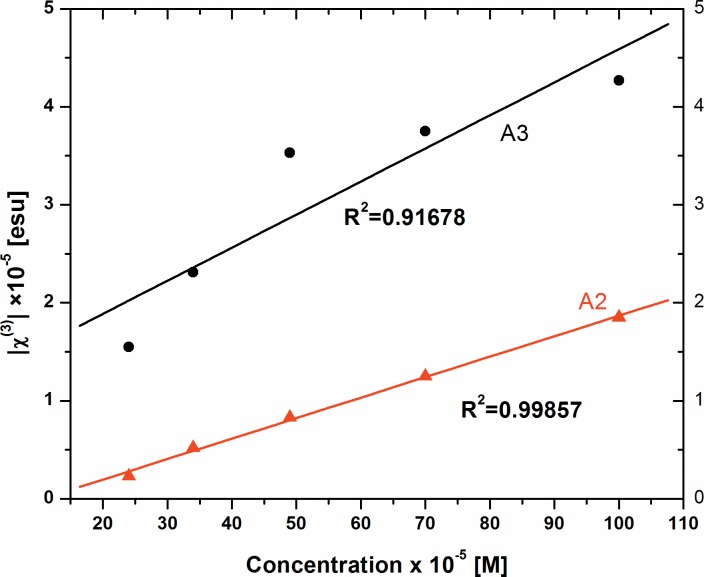
Variation of absolute third order susceptibility |*χ*^(3)^| of Dyes A2 and A3 as a function of concentration.

The evaluated values of |*χ*^(3)^| of dyes A2 and A3 are plotted in Fig 10 and tabulated in Table A in S3 File. The values reported are comparable with earlier reports [18].

**Fig 11:**
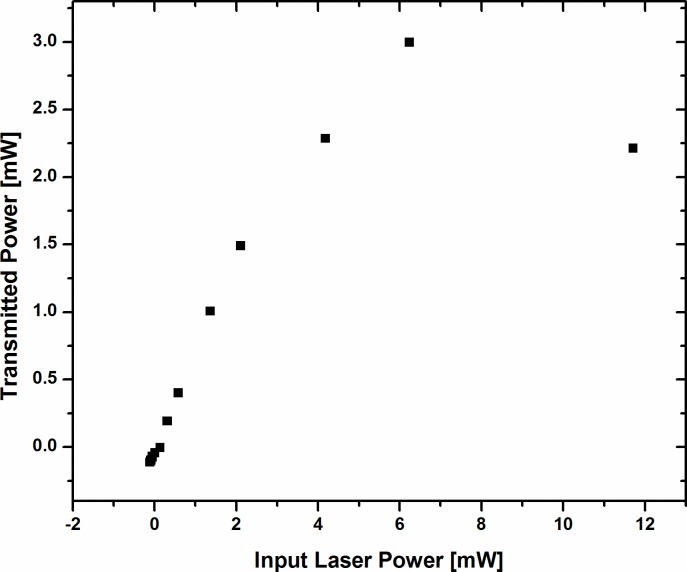
Variation of transmitted power with input laser power of dye A3, recorded at a fixed position (valley point) and at concentration 100x10^-5^ M.

We studied the optical limiting behavior of the dyes at concentration 100x10^-5^ M. The sample was kept at the valley position and the input laser power was varied using neutral density filters. The output transmittance with aperture closed is measured at different input laser powers. Fig 11 shows the data for dye A3, similar results are obtained for dye A2. As can be seen from the figure the transmitted power is progressively getting saturated and limited to about 3 mW, as the input laser power is increased to 12 mW. The trend shows the optical limiting behavior of the dye.

The high values of negative nonlinear refractive index and the third order susceptibility |***χ***^(**3**)^| of these dyes suggest that dyes A2 and A3 could be used as optical limiters to protect instrument and human eyes as well as have a potential application in nonlinear optical devices [51, 52], whereas dye A1 shows much smaller |***χ***^(**3**)^| and maybe unsuitable for such application.

### Density Functional Theoretical Study

As described in the experimental section above, the DFT calculations for the novel dyes structure were optimized, the shift in the spectra in different solvent environment and their nonlinear optical properties were studied using the Gaussian view software. The results obtained are discussed below.

#### Geometry

**Fig 12:**
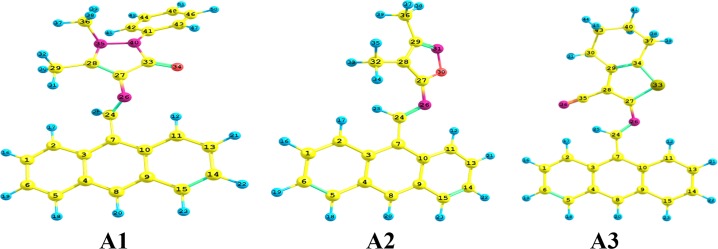
Selected bond lengths and angles of the optimized geometry of the molecules of A1, A2 and A3 compounds.

Selected bond lengths and angles of the optimized geometry of the elected molecules (A1, A2 and A3) (Fig 12) which have been estimated by applying CAM-B3LYP/6-311++G** level of theory are depicted in Table 4. The remarks drawn from Table 4 include: (1) the C7-C24 bond lengths for A1, A2 and A3 are shorter than expected by 0.058, 0.065 and 0.068Å, respectively, compared to that of ethane [53]; while their N26-C27 bond lengths are shorter by 0.084, 0.098 and 0.097Å, respectively, compared to methylamine [54]. This indicates that they have multiple bond nature and hence act as bridges for charge transfer passage. (2) The C7C24N26 and C24N26C27 angles of *ca*. 125° and 120°, respectively, show a sp^2^ hybridization scheme around the azomethine bridge connecting the anthracene and pentagonal rings. (3) The co-planarity between the anthracene ring and the -C = N- bridge for A1, A2 and A3 compounds are dictated by the angles C3C7N26C27 as 36°, 24° and 24°, respectively; while those for the azomethine bridge and the pentagonal ring are given by 60°, 45° and 39°, respectively. These findings denote that the order of co-planarity between these moieties as A3 > A2 > A1.

**Table 4:** Some selected bond lengths (Å) and bond and torsional angles (degrees) for gas-phase A1, A2 and A3 which have been calculated with CAM-B3LYP/6-311++G** level of theory.

Designation	A1	A2	A3
C7C24	1.474	1.467	1.464
C24N26	1.269	1.276	1.279
N26C27	1.390	1.376	1.377
C27C28	1.351	1.364	1.379
C7C24N26	124.1	125.5	125.8
C24N26C27	119.7	118.8	120.0
N26C27C28	130.6	134.4	131.7
C3C7C24N26	144.7	156.1	177.8
C24N26C27C28	59.7	45.4	39.0

#### Frontier Molecular Orbitals

**Fig 13:**
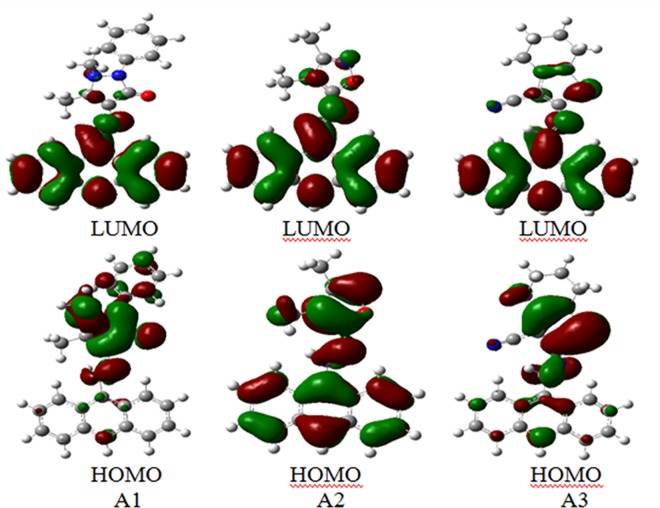
The frontier molecular orbitals (FMOs) (HOMOs and LUMOs) of A1, A2 and A3 compounds.

The Frontier Molecular Orbitals (FMOs) of gas-phase A1, A2 and A3 molecules are shown in Fig 13. They were estimated by applying CAM-B3LYP/6-31G* level of theory. The LUMOs of the three substrates are delocalized over the entire anthracene ring, as π-antibonding orbitals; while their HOMOs are dispersed amongst the pentagonal rings and the azomethine moieties as π-bonding orbitals and as lone pairs on the oxygen and sulphur atoms. These arrangements favour the intramolecular charge transfer from the pentagonal rings towards the anthracene rings across the azomethine bridges. In Table 5 are registered the energies of the HOMOs and LUMOs together with their energy gaps (E.G.) of the gas-phase A1, A2 and A3 substrates. The relative stabilities of the LUMOs of the studied compounds are A3 > A2 > A1; while the relative stabilities of their HOMOs are A2 > A3 > A1. As a result, A3 has the smallest energy gap (5.215 eV) while A1 has the highest one (5.590 eV). It is observed that their energy gaps of 5.215–5.590 eV indicate intramolecular charge transfer transitions of π→π* and n→π* nature. This is evidenced by the observed UV-Visible spectra.

**Table 5:** The ground state total zero-point electronic energy (E/au), the dipole moment (D.M./Debye), the HOMO (eV), LUMO (eV) the energy gap (E.G./eV), the electronic chemical potential (μ/eV), the chemical hardness (η/eV), the global electrophilicity index (ω/eV) and total hyperpolarizability (β_tot_/a.u.) for gas-phase A1, A2 and A3 using CAM-B3LYP/6-31G* level of theory. Their counterparts for p-nitroaniline (pNA) calculated by using CAM-B3LYP/6-31G* were listed for comparison purposes.

Parameter	A1	A2	A3	pNA^a*^	pNA^b*^
D.M.	4.811	5.086	2.216	7.304	6.9
LUMO	-0.839	-1.268	-1.298	-0.522	—
HOMO	-6.429	-6.673	-6.513	-7.468	—
E.G.	5.590	5.405	5.215	6.946	—
μ	-3.634	-3.971	-3.906	-3.995	—
η	2.795	2.703	2.608	3.473	—
ω	2.362	2.917	2.925	2.298	—
β_tot._	383	309	622	1176	1072±44

a* This work

b* Experimental values taken from Ref.60

In Table 5, we have also listed the gas-phase electronic chemical potential (μ) that exhibits the eloping proclivity of electrons in a chemical system [55], the chemical hardness (η) as a contributory motif for investigating stability and reactivity of chemical systems [56] and the global electrophilicity index (ω) that measures the stabilizing energy, when a chemical entity picks supplemental electronic charge from the medium [55]. It is clear from Table 5 that the μ values indicate that the order of chemical potential is A2<A3<A1; meaning that A1 is the most stable and least reactive while A2 is the least stable and most reactive amongst them. In the meantime, the η values show that the order of chemical hardness (stability or low reactivity) is A1>A2>A3. Therefore, A1 is the hardest (highly stable) while A3 is the softest (highly unstable) among them. In addition, the ω values denote that A3 is a strongest electrophile, while A1 is weakest electrophile or the strongest nucleophile among them [57].

Detailed studies of A1, A2 and A3 in DMSO, CH_3_OH, CH_2_Cl_2_, THF and CHCl_3_ are listed in Tables B, C and D in S3 File respectively. It is noticeable that the energy gaps (E.G.) and chemical hardnesses (η) of the solvated A1, A2 and A3 substrates are inversely proportional to their dielectric constants *i*.*e*. their polarities; while their chemical potentials (μ) and electrophilicity indices (ω) are directly proportional to them. These facts boost the intermolecular charge transfer between A1, A2 and A3 and the solvents applied.

#### UV-Visible Spectra

The π→π* and n→π* transitions in π-conjugated organic molecules are manifested by UV–Visible absorption spectra [58]. These transitions embrace electron motions between the higher occupied molecular orbitals (HOMOs) and the lower unoccupied molecular orbitals (LUMOs). The studied molecules A1, A2 and A3 have many double bonds together with lone pairs on the nitrogen, oxygen and sulphur atoms. The experimental and theoretical UV–Visible transitions of A1, A2 and A3 in DMSO, CH_3_OH, CH_2_Cl_2_, THF and CHCl_3_ are depicted in Table 6. The PCM method at TD-CAM-B3LYP/6-31G* level of theory was applied to predict the UV-Visible transitions in these solvents. In aggregate, a harmonization exists between the experimental and theoretical peaks in trend. On the one hand the red shifts and oscillator strengths of the predicted maximum emission wavelengths are directly proportional to the polarities of the solvents used. On the other hand, the red shifts and oscillator strengths of absorption wavelengths are proportional to the polarities of non-chlorinated solvents; while the chlorinated ones induced more red shifts and oscillator strengths in line with their polarities. Apart from A1 in chloroform, the excited states of all other solvated substrates are more polar than the ground states. The difference between the dipole moments of ground and excited states of the solvated substrates are proportional to the polarity of elected solvents. The inconsistency between the experimental and the theoretical wavelengths in magnitude could be due to the solvent effects *i*.*e*. the solute-solvent charge transfer complicates the chemical ambience of the molecules [59] and partially due to the absence of geometrical and vibrational relaxation terms in the calculations [60]. Computationally, the embodiment of dispersion corrections in the DFT functionals, in addition to using larger basis sets, could further bridge the gaps between them.

Detailed studies of A1, A2 and A3 in DMSO, CH_3_OH, CH_2_Cl_2_, THF and CHCl_3_ are listed in Tables B, C and D in S3 File respectively. It is noticeable that the energy gaps (E.G.) and chemical hardness (η) of the solvated A1, A2 and A3 substrates are inversely proportional to their dielectric constants *i*.*e*. their polarities; while their chemical potentials (μ) and electrophilicity indexes (ω) are directly proportional to them. These facts boost the intermolecular charge transfer between A1, A2 and A3 and the solvents applied.

The UV-Vis. observed and calculated wavelengths (λ/nm) for A1, A2 and A3 in different solvent and their transition energies (eV), oscillator strengths and assignments were facilitated by using TD-CAM-B3LYP/6-31G(d) level of theory in Table 6.

**Table 6:** The experimental and theoretical UV-Vis. maximum absorption (λ_abs_/nm) and emission (λ_em_/nm) wavelengths and their oscillator strengths(*f*); together with the ground (μ_G.S._/Debye) and excited (μ_ES._/Debye) dipole moments and their difference (Δμ/Debye) for the solvated dyes, which were calculated by using TD-CAM-B3LYP/6-31+G* level of theory.

Solvent	λ_abs_/nm		*f*	λ_em_/nm		*f*	μ_G.S._	μ_E.S._	Δμ
	Expt.	Theor.		Expt.	Theor.				
**Dye A1**									
DMSO	423	383.3	0.519	446	567.4	0.806	6.136	6.352	0.216
CH_3_OH	410	382.0	0.504	474	566.0	0.802	6.103	6.301	0.198
CH_2_Cl_2_	416	385.1	0.525	474	554.6	0.768	4.818	5.892	0.074
THF	415	383.3	0.516	472	551.7	0.759	5.742	5.787	0.045
CHCl_3_	415	384.2	0.522	473	542.5	0.729	5.498	5.454	-0.044
**Dye A2**									
DMSO	439	397.5	0.442	472	563.5	0.696	7.230	8.880	1.650
CH_3_OH	435	395.9	0.428	467	562.3	0.693	7.202	8.834	1.632
CH_2_Cl_2_	433	397.3	0.443	468	553.0	0.664	6.950	8.454	1.504
THF	429	396.9	0.441	467	550.5	0.656	6.884	8.355	1.471
CHCl_3_	435	397.4	0.447	468	542.6	0.631	6.669	8.035	1.368
**Dye A3**									
DMSO	438	413.3	0.561	483	657.9	0.836	3.236	3.847	0.611
CH_3_OH	425	411.6	0.547	497	656.5	0.832	3.221	3.821	0.600
CH_2_Cl_2_	439	414.1	0.566	483	645.4	0.799	3.101	3.608	0.507
THF	451	413.9	0.564	481	642.6	0.790	3.069	3.554	0.485
CHCl_3_	440	415.2	0.574	493	634.5	0.758	2.964	3.388	0.424

#### Natural Bond Orbital Analysis

Natural Bond Orbital (NBO) theory [60] is widely acceptable in investigating hyperconjugative interactions [61]. This is accomplished through applying second order perturbation energies (E_(2)_):

$$E_{2}={\Delta E}_{ij}=q_{i}(F_{ij}{)}^{2}/\Delta \varepsilon$$ (13)

where q_i_ is the occupancy of the donor orbital, F_ij_ is the NBO Kohn-Sham off-diagonal matrix elements and Δε is the energy difference between that of a donor orbital (i) and an acceptor orbital (j). The second order perturbation hyperconjugative energies (E_(2)_) of the gas-phase A1, A2 and A3 molecules, which were estimated by applying CAM-B3LYP/6-31G* level of theory, are registered in Table 7. They are assorted as π→π* and n→π* electronic transitions. The common intramolecular charge transfer from the pentagonal ring toward the anthracene ring and through the azomethine moiety is exemplified by the following interactions: (1) the strong π→π* electronic transitions between the C27-C33, C27-C28 and C24-N26 π-bonds with C24-N26, C24-N26 and C7-C10 π-antibonds, respectively. They stabilized A1, A2 and A3 compounds with totals of 17.53, 24.20 and 16.81 kcal/mole, respectively. (2) The weak n_N26_→π* hyperconjugative interactions contributed 3.58, 3.24 and 3.33 kcal/mole for the stabilization of A1, A2 and A3, respectively. (3) The extremely strong delocalization transitions between the oxygen and sulphur atoms lone pairs (n_O30_ and n_S33_) and the π-antibonds (C27-C28) in A2 and A3 availed 45.82 and 35.39 kcal/mole, respectively, for their stabilization. These results endorse our experimental UV–Visible spectra and adhere largely with our theoretical predictions mentioned above. Evidently, there are other strong π→π* and n→π* transitions which are uncommon between the three dyes. We neglected them, as they are unhelpful at this stage comparison.

**Table 7:** Some selected second order perturbation (E_(2)_) estimation of the hyperconjugative energies (kcal/mol) of A1, A2 and A3 which show the charge transfer from HOMO to the LUMO through the azomethine bridge. They were calculated using CAM-B3LYP/631G* level of theory.

Transition	A1	A2	A3
π_C33C27_→π*_C24N26_	5.98	8.73	0.77
π_C27C28_→π*_C24N26_	5.98	8.73	9.34
π_C24N26_→π*_C7C10_	5.57	6.74	6.70
n_N26_→π*_C7C24_	3.58	3.24	3.33
n_O30_(n_S33_)→π*_C27C28_	—	45.82	35.39

#### Nonlinear Optical Properties

There are a number of ways to estimate hyperpolarizability. Consequently, ambiguity often happens when observed and calculated data are matched [62]. In this paper, we estimate the total hyperpolarizability, β_tot_, by the equation:

$$\beta_{tot}=(\beta_{x}^{2}+\beta_{y}^{2}+\beta_{z}^{2}{)}^{1/2}$$ (14)

where

βi=βiii+13∑(βijj+βjij+βjji) (15)

The total hyperpolarizabilies of A1, A2 and A3 molecules registered in Table 5 are given in atomic units (a.u.) [1 a.u. = 8.6393X10^-33^ esu]. The calculated dipole moments (D.M.), and the HOMO-LUMO energy gaps (E.G.) which were computed by using CAM-B3LYP/6-31G* level of theory are listed also in Table 5. Para-nitroaniline (pNA), is considered as a reference of high charge-transfer entity and high hyperpolarizability, which has been obtained both experimentally [63, 64] and theoretically [65]. In rapprochement with pNA, we envisage that the three gas-phase dyes A1, A2 and A3 have commensurable non-linear optical (NLO) properties, with that of A1 equalizing to 33%, A2 to 26% and A3 to 53% that of pNA calculated by CAM-B3LYP/6-31G* level of theory.

Table 5 also registers the energy gaps (E.G.) for the three gas-phase dyes A1, A2 and A3. A number of calculated [65, 66] and observed [67] investigations show an inverse relation between the total hyperpolarizabilities and the energy gaps. This yardstick expedites the probability of charge transfer that procures to higher hyperpolarizability. Our monitored dyes breach this linkage. This is because many other instruments transcribe the consolidated hyperpolarizabilities. They encompass, in addition to, small energy gaps, planarity, large dipole moments, presence of H-bonding and a push-pull mechanism [36, 68, 69]. Both A2 and A3 are more planar than A1; while the latter has higher both dipole moment and energy gap. The total hyperpolarizabilities of A1 and A2 are comparable, while that of A3 is *ca*. twice of them. This is, probably, because it has lower energy gap and a comparatively higher co-planarity between the anthracene ring, the azomethine linkage and the pentagonal ring. The competiveness of A1 over A2, despite having a larger band gap and a lesser co-planarity, could be due the strong π→π* and n→π* transitions between the pentagonal moiety and the benzene ring. These findings could substantiate the experimental results without being in quantitative agreement, as it is widely known that the comparison between the theoretical and measured hyperpolarizabilities is not straightforward [70–72].

In solution, the picture has changed drastically *i*.*e*. the order of the total hyperpolarizabilities became A1 > A2 > A3. In addition, the total hyperpolarizabilities of solvated A1, A2 and A3 are directly proportional to the polarity of solvents. Their values are 4–7, 4–5 and 2 times greater than those for their gas-phase counterparts, respectively, in good agreement with the that of pNA in methanol using ωB97XD/6-311+G** level of theory [73]; which is enhanced by nearly a factor of four compared to its gas-phase value.

## Conclusions

The concentration dependent nonlinear refractive index, nonlinear absorption and third order susceptibility were studied for the novel dyes A1, A2 and A3. The measured value of all these nonlinear optical properties reveals that the dyes A2 and A3 exhibit large optical nonlinearity whereas the dye A1 has relatively lower nonlinearity. These experimental results show that dyes A2 and A3 can be used as promising materials for application in nonlinear optical devices. In addition, HOMO, LUMO, energy gap (ΔE), chemical potential (μ), chemical hardness (η), electrophilicity index (ω) and total hyperpolarizability (β_tot_), supported by natural bond orbital technique, were investigated using density functional theory. It is concluded, computationally, that molecule A1 is the hardest molecule but the weakest electrophile, while A3 is the softest compound but the strongest electrophile amongst them. In gas-phase, the total hyperpolarizabilities of A1 and A2 are comparable, while that of A3 is *ca*. twice of them. In solution,the energy gaps of solvated A1, A2 and A3 dyes are inversely proportional to their polarities. Consequently, this facilitates the intermolecular charge transfer phenomenon and resulted in enhancing the total hyperpolarizabilities by a factor of 4–7, 4–5 and 2 for A1, A2 and A3, respectively, compared to their gas-phase values.

## Supporting Information

S1 File Synthesis and characterization of the novel dyes A1, A2 and A3. (DOCX)

S2 File **Fig A,** Electronic absorption spectra of 1 × 10^−5^ M of compound A1 in different solvents**. Fig B,** Emission spectra of 1 × 10^−5^ M of compound A1 in different solvents. **Fig C,** Electronic absorption spectra of 1 × 10^−5^ M of compound A2 in different solvents. **Fig D,** Emission spectra of 1 × 10^−5^ M of compound A2 in different solvents. **Fig E,** Electronic absorption spectra of 1 × 10^−5^ M of compound A3 in different solvents. **Fig F,** Emission spectra of 1 × 10^−5^ M of compound A3 in different solvents.
(DOCX)

S3 File **Table A,** Nonlinear refractive index, nonlinear susceptibility and nonlinear absorption at different concentration for Dyes A2 and A3. **Table B,** DFT calculated parameters for dye A1 in different solvents. **Table C,** DFT calculated parameters for dye A2 in different solvents. **Table D,** DFT calculated parameters for dye A3 in different solvents.
(DOCX)
