# Supplementary material for: Physicochemical and Nonlinear Optical Properties of Novel Environmentally Benign Heterocyclic Azomethine Dyes: Experimental and Theoretical Studies
Source: PLoS One. 2016 Sep 15;11(9):e0161613. doi: 10.1371/journal.pone.0161613 (PMC5025016; doi:10.1371/journal.pone.0161613)
Supplement: S1 File — (DOCX) [file pone.0161613.s001.docx]

1. **Synthesis and characterization of the novel dyes A1, A2 and A3**

### 4-[(Anthracen -9-ylmethylene)-amino]-1, 5-dimethyl-2-phenyl-1,2-dihydro-pyrazol-3-one (A1)

Red-yellow solid: Yield: 90%; m.p. 232 °C; GC-MS m/z (rel. int.%): 393 (68) [M+1]^+^; IR (KBr) *v*_max_ cm^-1^: 3027 (Ar-H, stretch), 2874 (C-H), 1636 (C=O), 1580 (C=N), 1138 (C-N); ^1^H NMR (600MHZ, CDCl_3_) (δ/ppm): 11.06 (s, CH olefinic), 8.98 (d, 1H, CH aromatic, J=8.84 Hz), 8.50 (d, 1H, CH aromatic, J=7.4 Hz), 8.04 (dd, 1H, CH aromatic, dd, J= 7.6 Hz, ), 7.50 (dd, 1H, CH aromatic, J=7.2Hz), 7.48 (s, 1H, CH aromatic), 7.56-7.51 (m, 5H, CH), 3.23 (s, 3H, CH_3_), 2.19 (s, 3H, CH_3_); ^13^CNMR ( CDCl_3_) δ: 160.84 (C=O), 157.70, 152.03, 134.75, 131.54, 130.45, 129.32, 129.01, 128.8, 127.11, 126.60, 125.64, 125.22, 124.62, 119.70 (C-aromatic), 35.78, 10.36; Anal. calc. for C_26_H_21_N_3_O: C, 79.77, H, 5.41, N, 10.73. Found: C, 79.74, H, 5.38, N, 10.68.

### Anthracen-9-ylmethlene-(3, 4-dimethyl-isoxazol-5-yl)-amine (A2)

Yellow solid: Yield: 86%; m.p. 146-147 °C; GC-MS m/z (rel. int.%): 301 (62) [M+1]^+^; IR (KBr) *v*max cm^-1^: 2917 (C-H), 1580 (C=N), 1158 (C-N); ^1^H NMR (600MHZ, CDCl_3_) (δ/ppm): 10.12 ( s, CH olefinic), 8.95 (d, 2H, CH aromatic, J=8.8 Hz), 7.61 (dd, 1H, CH aromatic, J=5.6 Hz), 7.50 (dd, 1H, CH aromatic, J=7.2 Hz), 8.56 (d, 1H, CH aromatic, J=8.0 Hz), 7.65 (s, 1H, CH aromatic), 2.30 (s, 3H, CH_3_), 2.16 (s,3H, CH_3_); ^13^CNMR ( CDCl_3_) δ: 165.44 (C=O), 162.07, 159.82, 132.63, 131.53, 131.10, 130.05, 129.27, 129.12, 127.94, 125.54, 124.07, 123.56 (C-Aromatic), 116.50, 108.03, 10.83, 6.89; Anal. calc. for C_20_H_16_N_2_O: C, 79.98, H, 5.37, N, 9.33. Found: C, 79.94, H, 5.32, N, 9.28.

### 2-[(Anthracen-9-ylmethylene)-amino]-4,5,6,7-tetrahydro-benzo[b] thiophene-3-carbonitrile (A3)

Dark brown: Yield: 88 %; m.p. 281°C; GC-MS m/z (rel. int.%): 368 (56) [M+1]^+^; IR (KBr) *v*max cm^-1^: 2935 (C-H), 1557 (HC=N), 1133 (C-N); ^1^H NMR (600MHZ, CDCl_3_) (δ/ppm): 9.76 ( s, CH olefinic), 9.05-7.52 (m, 9H, CH aromatic), 2.77-1.89 (m, 8H, -CH_2_) ^13^CNMR ( CDCl_3_) δ: 161.00 (C=O), 157.75, 135.37, 133,25, 132.61, 131.81, 131.29, 129.37, 129.30, 128.37, 125.62, 124.72, 124.32 (C-Aromatic), 114.71, 106.68, 43.45, 25.34, 24.40, 23.12, 22.05; Anal. calc. for C_24_H_18_N_2_O: C, 78.66, H,4.95, N, 7.64. Found: C, 78.62, H, 4.88, N, 7.58.
