# Supplementary material for: Physicochemical and Nonlinear Optical Properties of Novel Environmentally Benign Heterocyclic Azomethine Dyes: Experimental and Theoretical Studies
Source: PLoS One. 2016 Sep 15;11(9):e0161613. doi: 10.1371/journal.pone.0161613 (PMC5025016; doi:10.1371/journal.pone.0161613)
Supplement: S2 File — Fig A, Electronic absorption spectra of 1 × 10^−5^ M of compound A1 in different solvents. Fig B, Emission spectra of 1 × 10^−5^ M of compound A1 in different solvents. Fig C, Electronic absorption spectra of 1 × 10^−5^ M of compound A2 in different solvents. Fig D, Emission spectra of 1 × 10^−5^ M of compound A2 in different solvents. Fig E, Electronic absorption spectra of 1 × 10^−5^ M of compound A3 in different solvents. Fig F, Emission spectra of 1 × 10^−5^ M of compound A3 in different solvents. (DOCX) [file pone.0161613.s002.docx]

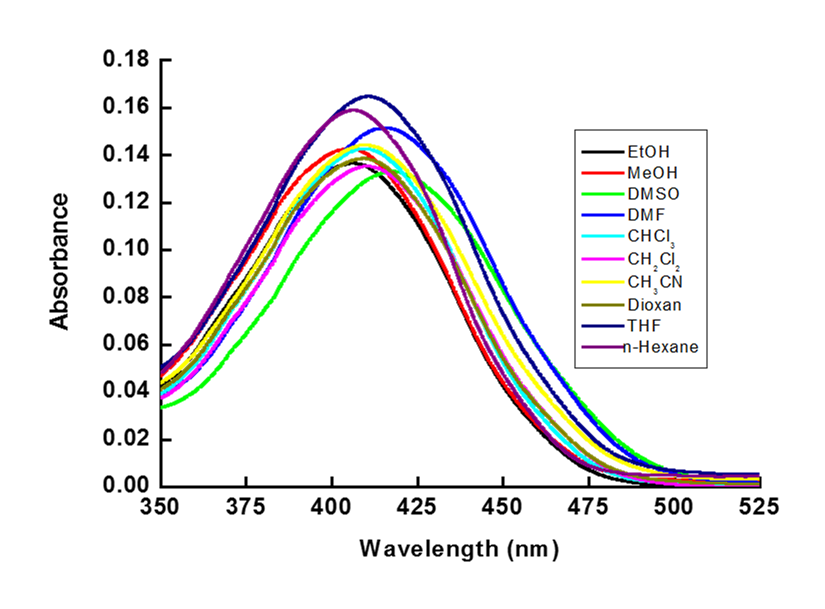


**S2 Figure A-** Electronic absorption spectra of 1 × 10^-5^ M of compound A1 in different solvents**.**


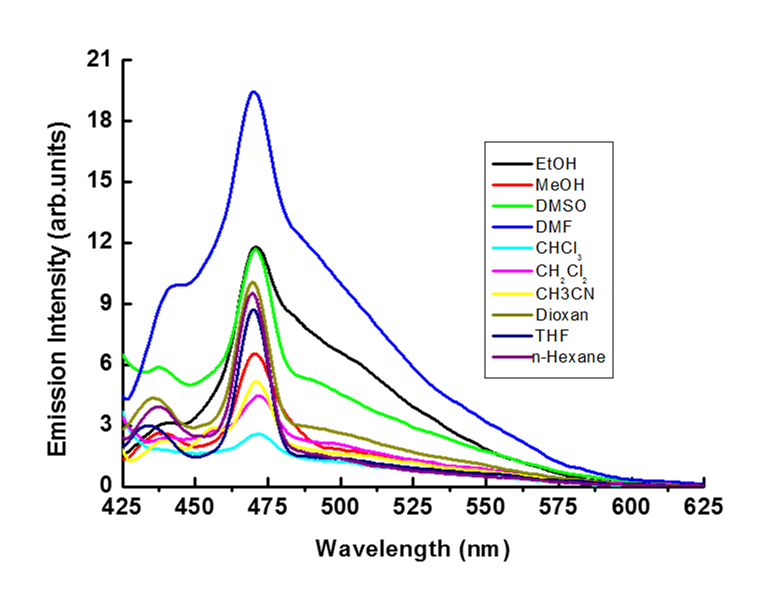


**S2 Figure B-** Emission spectra of 1 × 10^-5^ M of compound A1 in different solvents


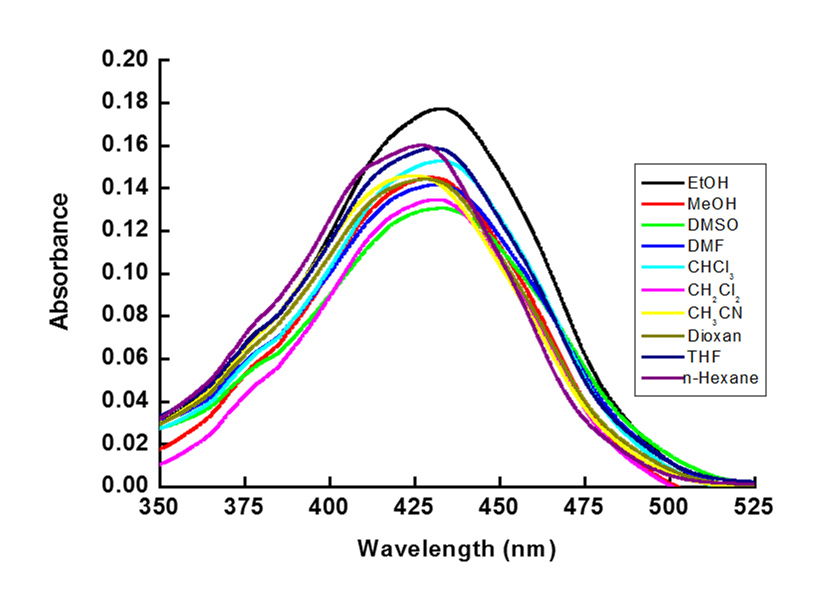


**S2 Figure C.**  Electronic absorption spectra of 1 × 10^-5^ M of compound A2 in different solvents


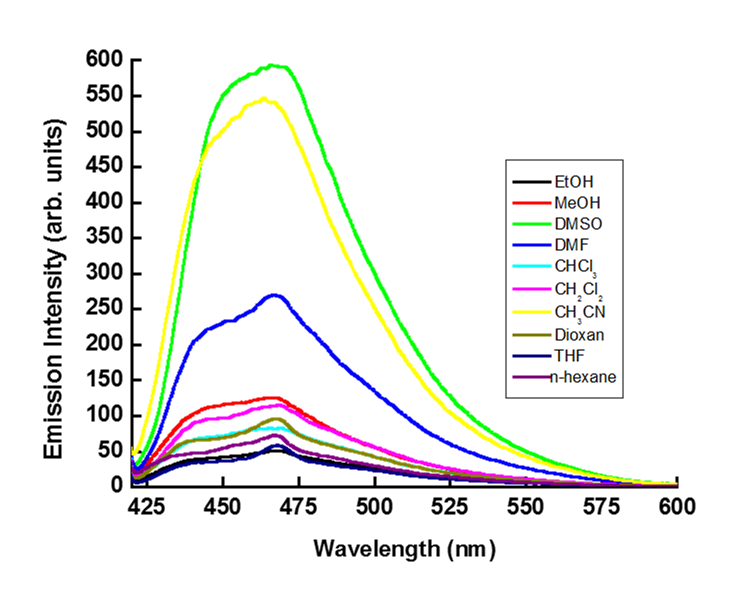


**S2 Figure D.** Emission spectra of 1 × 10^-5^ M of compound A2 in different solvents


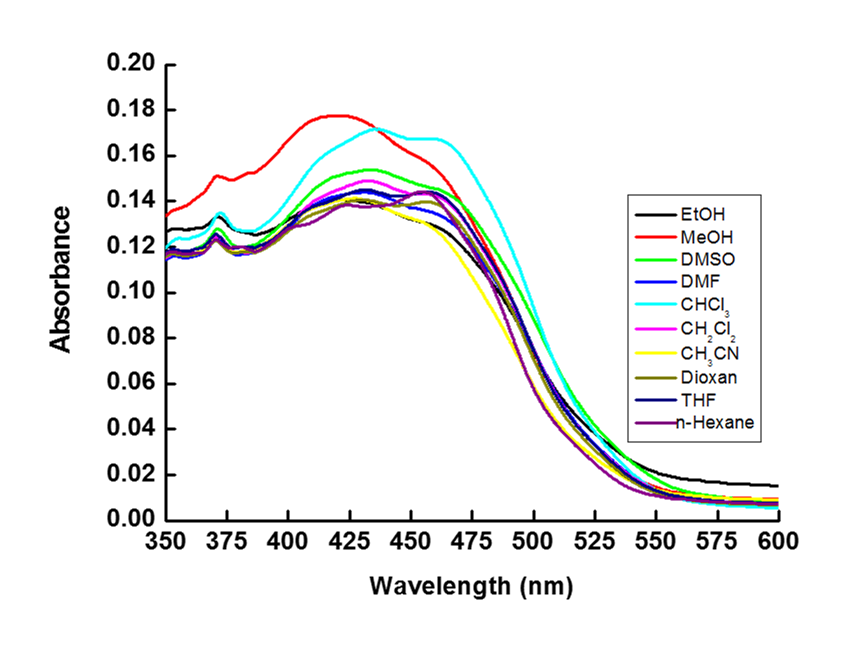


**S2 Figure E.**  Electronic absorption spectra of 1 × 10^-5^ M of compound A3 in different solvents


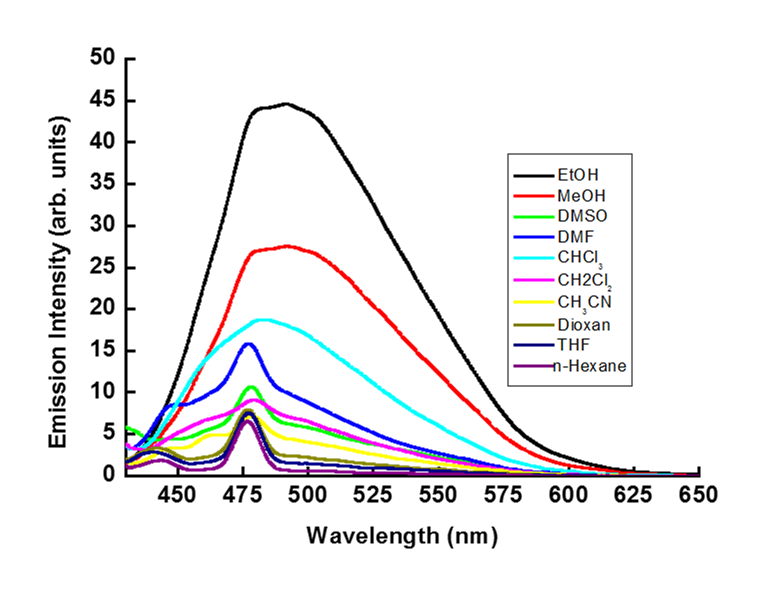


**S2 Figure F.**  Emission spectra of 1 × 10^-5^ M of compound A3 in different solvents
