# Supplementary material for: Physicochemical and Nonlinear Optical Properties of Novel Environmentally Benign Heterocyclic Azomethine Dyes: Experimental and Theoretical Studies
Source: PLoS One. 2016 Sep 15;11(9):e0161613. doi: 10.1371/journal.pone.0161613 (PMC5025016; doi:10.1371/journal.pone.0161613)
Supplement: S3 File — Table A, Nonlinear refractive index, nonlinear susceptibility and nonlinear absorption at different concentration for Dyes A2 and A3. Table B, DFT calculated parameters for dye A1 in different solvents. Table C, DFT calculated parameters for dye A2 in different solvents. Table D, DFT calculated parameters for dye A3 in different solvents. (DOCX) [file pone.0161613.s003.docx]

**SUPPLIMENTARY TABLES:**

**S3 Table A.** Nonlinear refractive index, nonlinear susceptibility and nonlinear absorption at different concentration for Dyes A2 and A3

|  | Dye A3 | | | Dye A2 | | |
| --- | --- | --- | --- | --- | --- | --- |
| CONC  ×10^-5^ [M] | n_2_ ×10^-7^  [cm^2^/W] | β ×10^-3^  [cm/W] | $\left\vert\chi^{(3)} \right\vert$×10^-5^  [esu] | n_2_ ×10^-7^  [cm^2^/W] | β ×10^-4^  [cm/W] | $\left\vert\chi^{(3)} \right\vert$ ×10^-5^  [esu] |
| 100 | -8.07 | 6.42 | 4.27 | -3.49 | 12.7 | 1.85 |
| 70 | -7.08 | 5.80 | 3.75 | -2.37 | 8.4 | 1.25 |
| 49 | -6.67 | 1.96 | 3.53 | -1.57 | 6.7 | 0.83 |
| 34 | -4.37 | 1.19 | 2.31 | -0.98 | 4.9 | 0.52 |
| 24 | -2.92 | 0.22 | 1.55 | -0.43 | 1.4 | 0.23 |

**S3 Table B.** The total zero-point electronic energy (au), relative energy with respect to that of DMSO (∆E/kcal/mol), the dipole moment (D.M./Debye), the LUMO (eV), HOMO (eV) the energy gap (E.G./eV), the electronic chemical potential (µ/eV), the chemical hardness (η/eV) and the global electrophilicity index (ω/eV) and total hyperpolarizability (β_tot_/a.u.) for A1 in different solvents. They have been calculated using TD-CAM-B3LYP/6-31G* level of theory. The Dielectric Constants (ε) of these solvents were given to indicate their polarity.

| **Parameter** | **DMSO** | **CH_3_OH** | **CH_2_Cl_2_** | **THF** | **CHCl_3_** |
| --- | --- | --- | --- | --- | --- |
| Energy | -1241.70331 | -1241.70286 | -1241.69926 | -1241.69832 | -1241.69540 |
| ∆E | 0.000 | 0.282 | 2.541 | 3.131 | 4.964 |
| D.M. | 9.166 | 9.055 | 8.169 | 7.955 | 7.342 |
| LUMO | -1.503 | -1.497 | -1.456 | -1.446 | -1.415 |
| HOMO | -5.865 | -5.862 | -5.848 | -5.844 | -5.832 |
| E.G. | 4.362 | 4.365 | 4.392 | 4.398 | 4.417 |
| µ | -3.684 | -3.680 | -3.652 | -3.645 | -3.624 |
| η | 2.181 | 2.183 | 2.196 | 2.199 | 2.209 |
| ω | 3.111 | 3.102 | 3.037 | 3.021 | 2.973 |
| ε | 46.83 | 33.1 | 8.93 | 7.43 | 4.71 |
| β_tot_ | 2504 | 2472 | 2183 | 2110 | 1532 |

**S3 Table C.** The total zero-point electronic energy (au), relative energy with respect to that of DMSO (∆E/kcal/mol), the dipole moment (D.M./Debye), the LUMO (eV), HOMO (eV) the energy gap (E.G./eV), the electronic chemical potential (µ/eV), the chemical hardness (η/eV) and the global electrophilicity index (ω/eV) and total hyperpolarizability (β_tot_/a.u.) for A2 in different solvents. They have been calculated using TD-CAM-B3LYP/6-31G* level of theory. The Dielectric Constants (ε) of these solvents were given to indicate their polarity.

| Parameter | DMSO | CH_3_OH | CH_2_Cl_2_ | THF | CHCl_3_ |
| --- | --- | --- | --- | --- | --- |
| Energy | -955.53244 | -955.53205 | -955.52887 | -955.52807 | -955.52555 |
| ∆E | 0.00 | 0.245 | 2.240 | 2.741 | 4.321 |
| D.M. | 8.466 | 8.402 | 7.890 | 7.762 | 7.359 |
| LUMO | -1.918 | -1.915 | -1.893 | -1.887 | -1.870 |
| HOMO | -6.346 | -6.345 | -6.335 | -6.332 | -6.324 |
| E.G. | 4.428 | 4.430 | 4.442 | 4.445 | 4.454 |
| µ | -4.132 | -4.130 | -4.114 | -4.110 | -4.097 |
| η | 2.214 | 2.215 | 2.221 | 2.223 | 2.227 |
| ω | 3.856 | 3.850 | 3.810 | 3.799 | 3.769 |
| ε | 46.83 | 33.1 | 8.93 | 7.43 | 4.71 |
| β_tot_ | 1692 | 1682 | 1436 | 1375 | 1188 |

**S3 Table D.** The total zero-point electronic energy (au), relative energy with respect to that of DMSO (∆E/kcal/mol), the dipole moment (D.M./Debye), the LUMO (eV), HOMO (eV) the energy gap (E.G./eV), the electronic chemical potential (µ/eV), the chemical hardness (η/eV) and the global electrophilicity index (ω/eV) and total hyperpolarizability (β_tot_/a.u.) for A3 in different solvents. They have been calculated using TD-CAM-B3LYP/6-31G* level of theory. The Dielectric Constants (ε) of these solvents were given to indicate their polarity.

| Parameter | DMSO | CH_3_OH | CH_2_Cl_2_ | THF | CHCl_3_ |
| --- | --- | --- | --- | --- | --- |
| Energy | -1432.05181 | -1432.05147 | -1432.04873 | -1432.04803 | -1432.04581 |
| ∆E | 0.000 | 0.213 | 1.933 | 2.372 | 3.765 |
| D.M. | 2.753 | 2.736 | 2.597 | 2.562 | 2.455 |
| LUMO | -2.106 | -2.102 | -2.074 | -2.067 | -2.048 |
| HOMO | -6.170 | -6.167 | -6.148 | -6.143 | -6.126 |
| E.G. | 4.064 | 4.065 | 4.074 | 4.076 | 4.078 |
| µ | -4.138 | -4.135 | -4.111 | -4.105 | -4.087 |
| η | 2.032 | 2.033 | 2.037 | 2.038 | 2.039 |
| ω | 4.213 | 4.205 | 4.148 | 4.134 | 4.096 |
| ε | 46.83 | 33.1 | 8.93 | 7.43 | 4.71 |
| β_tot_ | 1448 | 1433 | 1311 | 1280 | 1186 |
